# Supplementary material for: Comprehensive Annotation and Functional Exploration of MicroRNAs in Lettuce
Source: Front Plant Sci. 2021 Dec 24;12:781836. doi: 10.3389/fpls.2021.781836 (PMC8739914; doi:10.3389/fpls.2021.781836)
Supplement: Supplementary file 3 [file Data_Sheet_9.ZIP › GSM2230753.plot/Lsa-miRN1664_Lsat_1_v5_gn_0_20061.1_365_TPlot.pdf]

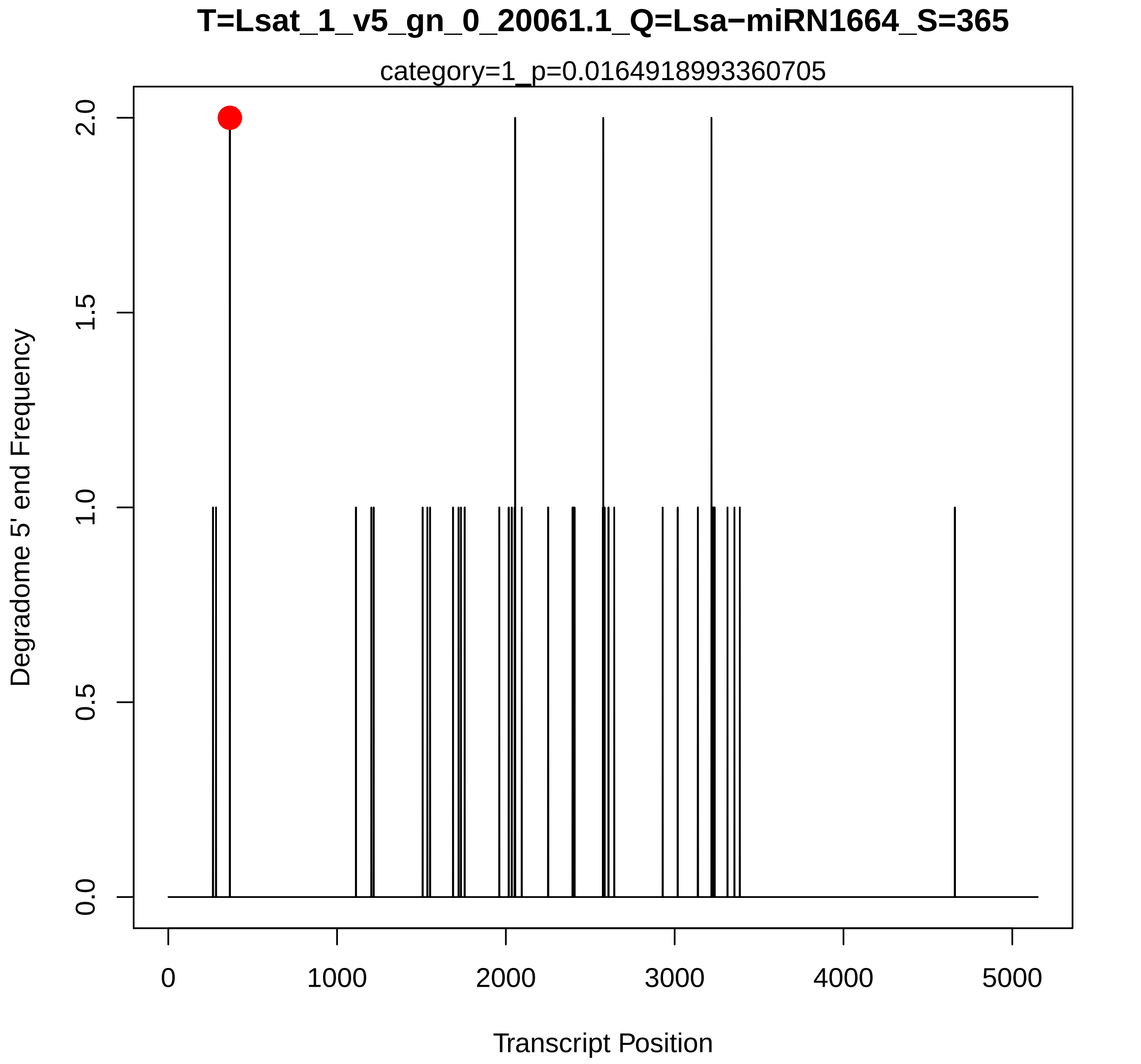

T=Lsat_1_v5_gn_0_20061.1_Q=Lsa−miRN1664_S=365
category=1_p=0.0164918993360705
Degradome 5' end Frequency
0.0
0.5
1.0
1.5
2.0
0
1000
2000
3000
4000
5000
Transcript Position
